# Incidental Finding of Post-Transplant Erythrocytosis After Renal Transplantation in a Patient With Chronic Kidney Disease: A Case Report

**DOI:** 10.7759/cureus.51218

**Published:** 2023-12-28

**Authors:** Nouman Anthony, Imran Khan, Abdullah Shah, Anum Tariq, Mudassar Khan

**Affiliations:** 1 General Medicine, Rehman Medical Institute, Peshawar, PAK; 2 Urology, Royal London Hospital, London, GBR; 3 Cardiothoracic Surgery, St. Bartholomew's Hospital, London, GBR; 4 Nephrology, Rehman Medical Institute, Peshawar, PAK; 5 Emergency Medicine, Rehman Medical Institute, Peshawar, PAK

**Keywords:** elevated erythropoietin, hematocrit (hct), polycythemia in pakistan, renal transplant surgery, post-transplant erythrocytosis

## Abstract

Renal transplant aims to provide a healthy substitute for the chronically damaged kidney while also correcting the anemia of chronic disease by producing erythropoietin for effective erythropoiesis. However, in a small number of renal transplant patients, the hematocrit continues to rise even after correction of the anemia, ultimately leading to abnormally increased hemoglobin and hematocrit. This condition is termed “post-transplant erythrocytosis” (PTE). We present a case of a 50-year-old male who was diabetic, positive for hepatitis B surface antigen, and negative for polymerase chain reaction. He presented with symptoms of acute hepatitis. During the work-up, PTE was diagnosed. Our case sheds light on a common complication of renal transplant known as PTE, its possible complications in the patient, and the necessary interventions to prevent untoward outcomes. PTE, although a less common complication of renal transplant, can become serious and potentially fatal due to its sequelae of thromboembolism. The complications can range from simple thrombophlebitis and thrombosis of digital and brachial arteries to more severe events such as pulmonary embolism or stroke and cardiovascular events. Regular post-transplant follow-ups with frequent bloodwork will aid in the early diagnosis of PTE, allowing for timely intervention with appropriate treatment options such as venesection or angiotensin receptor blockers (ARBs)/angiotensin-converting enzyme (ACE) inhibitors.

## Introduction

Post-transplant erythrocytosis (PTE) is a relatively uncommon complication, occurring in 8-15% of all kidney transplantations. PTE is a persistent elevation of hemoglobin (Hb) >17 g/dL or hematocrit (Hct) levels >51% following a kidney transplant [1].

PTE most commonly develops within 8-24 months after the renal transplant. However, the level of steady-state Hct depends on graft efficiency and the type of immunosuppression used [2]. Although few studies have been conducted on PTE, the available literature suggests several risk factors for developing PTE. These include male gender, normal Hb/Hct levels before the transplant, previous nephrectomy, renal artery stenosis, patients with efficiently working grafts, and patients who underwent hemodialysis before transplantation. If a native kidney with stenosed vessels is left in place, it also increases erythropoietin (EPO) production. Similarly, a patient with adequate EPO levels before transplant will have a higher chance of developing PTE compared to those who are taking recombinant human EPO [2]. Around 60% of patients with PTE show symptoms of malaise, headache, plethora, lethargy, and dizziness. Due to increased viscosity, there is an increased risk of stroke and thromboembolic events such as pulmonary embolism, deep vein thrombosis, and myocardial infarction, and 1% to 2% eventually die of associated complications [2,3].

Even though the mechanism of PTE development remains unclear, effective therapies are available that can readily address this complication of renal transplant. Earlier diagnosis and management with angiotensin-converting enzyme inhibitors (ACEIs)/angiotensin receptor blockers (ARBs) has shown better outcomes related to PTE.

## Case presentation

**Figure 1:**
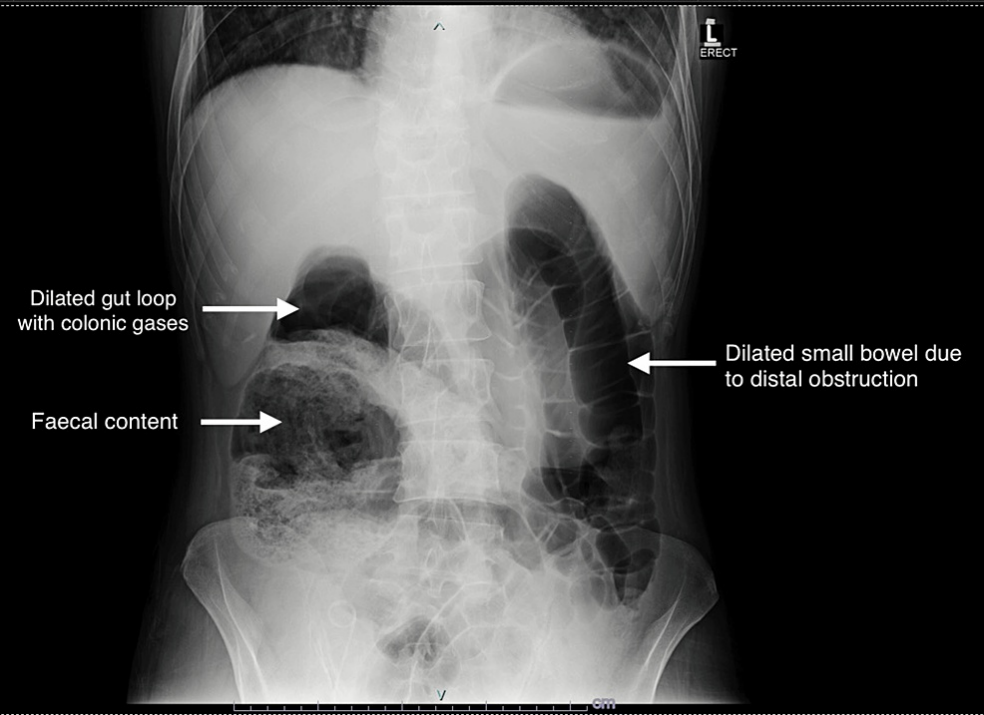
Abdominal X-ray showing dilated gut loops with colonic gases and fecal content on the right side along with dilated small bowel due to distal obstruction on the left side.

A 50-year-old male who underwent a renal transplant for chronic kidney disease with end-stage renal disease (ESRD) three months ago presented to the Emergency Room with complaints of abdominal discomfort, loss of appetite, constipation, and right hypochondriac pain over the past week. He was diabetic, non-hypertensive, positive for hepatitis B surface antigen, negative for polymerase chain reaction, and was taking entecavir 0.5 milligrams on alternate days. On examination, the patient was well-oriented and comfortable. His vital signs were as follows: blood pressure of 130/75 millimeters of mercury (mmHg), pulse rate of 88 beats per minute, respiratory rate of 12 breaths per minute, and temperature of 37°C. The general physical examination did not reveal any notable findings. Abdominal examination showed slight tenderness over the right hypochondriac region, with no organomegaly and negative shifting dullness. Laboratory workup revealed an Hb level of 16.2 g/dL, total leucocyte count of 23.12 x 10^9^/L, and platelet count of 263 x 10^9^/L. His tacrolimus level was 5.4 ng/mL, creatinine was 1.0 mg/dL, sodium was 129.4 mmol/L, and potassium was 5.04 mmol/L. Urine routine examination showed +1 protein, 2-4 pus cells, and 6-8 red blood cells (RBCs) (the RBC membranes were not examined). In the gastrointestinal profile, alanine aminotransferase was 224 international units per liter (IU/L), lactate dehydrogenase was 225 IU/L, serum amylase 25 IU/L, serum lipase 10 IU/L, and bilirubin was 0.8 milligrams per deciliter (mg/dL). Serology for cytomegalovirus, hepatitis A, hepatitis C, and hepatitis E were negative. Abdominal ultrasound revealed grade 3 parenchymal disease in the native kidney, while the transplanted kidney in the right hemipelvis showed normal indices. No ascites were seen on ultrasound, and the liver exhibited a hyperechoic texture due to fatty deposition. Abdominal X-ray demonstrated dilated gut loops with colonic gases and fecal content in the ascending colon along with a dilated small bowel due to distal obstruction on the left side (Figure 1).

After consultation with a gastroenterologist, the patient was advised a Kleen enema, after which the patient passed stool. Following 5 days of hospital management with IV ceftriaxone and fluids, the patient was discharged with the following medications: capsule tacrolimus 0.5 mg once a day (OD), prednisolone 5 mg OD, entecavir 0.5 mg OD, famotidine 40 mg OD, nystatin drops 100,000 IU OD, and aspirin 75 mg OD. When the patient presented for a follow-up after four weeks, there were no active complaints, and the liver enzymes had returned to normal levels. However, the complete blood count (CBC) showed surprisingly elevated Hb and Hct levels.

In the subsequent monthly follow-ups, the Hb and Hct levels continued to rise, which was unique because the patient was not currently taking any supplements such as iron or recombinant human EPO. Six months after his renal transplant, the patient began experiencing malaise, plethora, and dizziness, which could be attributed to his elevated Hb level of 18.5 g/dL and Hct level of 59.4%. During subsequent follow-ups, the patient's Hb and Hct levels continued to rise. These laboratory findings were accompanied by clinical symptoms of malaise, plethora, and dizziness, ultimately leading to the diagnosis of PTE. The monthly chart of the patient's Hb and Hct levels can be seen in Table 1.

**Table 1:** Monthly chart of the patient's Haemoglobin and Haematocrit levels.

Date of testing	Hemoglobin (g/dL) (normal: 12.5-16.5 g/dL)	Hematocrit (%) (normal: 35-50%)
January 23, 2022 (at the time of transplant, pre-op)	11.2 g/dL	39.5%
March 18, 2022	14.3 g/dL	45.5%
April 10, 2022	15.1 g/dL	48.4%
May 4, 2022 (at the time of presentation to the Emergency Room)	16.4 g/dL	49.6%
June 3, 2022	17.1 g/dL	54.3%
August 6, 2022	18.5 g/dL	59.4%

Considering the latest Hb and Hct levels, the diagnosis of PTE was established. The patient's EPO assay was conducted to support the diagnosis, revealing an EPO level of 22.8 million units per milliliter (mIU/mL). The patient was advised to undergo intermittent venesection to achieve a target Hct of 50-52%. Following that, he was initiated on losartan 50 mg OD, with recommended monthly CBC screening.

## Discussion

One out of seven patients living with chronic kidney disease suffer from anemia, commonly referred to as anemia of renal disease, which accounts for approximately 15% of all CKD patients [3]. The etiology and pathogenesis of this anemia can be primarily attributed to severely reduced production and ultimately very low serum EPO levels in CKD patients [4], as peritubular cells of the kidney remain the primary source of EPO production [5]. A similar issue was observed in our patient before his renal transplant, with a pre-operative Hb level of 11.2 g/dL, despite using recombinant human EPO. The definitive treatment for ESRD is a renal transplant, with dialysis being one of its modalities. In our case, the patient was young and a donor was available, leading the patient to undergo a kidney transplant. The patient's immunosuppressive regimen included tacrolimus as the primary choice of agent [6,7].

The patient's full blood count (FBC) showed an Hb level of 15.6 g/dL and Hct of 49.7%, which was surprisingly better than the estimated rise in Hb levels after the transplant (usually Hb levels rise to 12 g/dL three months post-transplant) [8]. As the patient's health improved after the enema and antibiotic treatment, he was discharged with prescribed medications and advised to follow up after four weeks. As mentioned earlier, PTE is associated with several risk factors, some of which were positive in our patients. Firstly, his male gender remains a significant risk factor. In a study conducted in Peshawar, Pakistan, it was observed that 64.4% of PTE patients were male [9]. Secondly, his non-undergoing nephrectomy for his native kidney further increased the risk of developing PTE. The patient's EPO assays were notably high at 22.8 mIU/mL, closely resembling the average EPO assay levels of 23.7 mIU/mL seen in patients with secondary erythrocytosis [10].

PTE signifies a hypercoagulable state that poses a considerable threat if it results in thromboembolism. To prevent such severe complications, the patient was started on losartan. ACEI or ARBs are the preferred first-line treatments for PTE, [11] as the renin-angiotensin-aldosterone system (RAAS) significantly influences EPO secretion from the renal parenchyma, with angiotensin-II increasing plasma EPO levels [12]. The patient was also advised intermittent phlebotomy for symptomatic relief; however, it does not confer any long-term benefits [13].

## Conclusions

In summary, a renal transplant performed for ESRD can lead to an uncommon complication of erythrocytosis within one to two years after the transplant. PTE can be identified earlier through frequent monitoring of the FBC and counselling the patient about being vigilant for symptoms such as plethora, malaise, and dizziness. ARBs or ACEIs remain the treatment of choice to prevent thromboembolism-related complications.
